# Chest ultrasonography in patients with HIV: a case series and review of the literature

**DOI:** 10.1007/s15010-015-0780-z

**Published:** 2015-05-14

**Authors:** Charlotte C. Heuvelings, Sabine Bélard, Saskia Janssen, Claudia Wallrauch, Martin P. Grobusch, Enrico Brunetti, Maria Teresa Giordani, Tom Heller

**Affiliations:** Center of Tropical Medicine and Travel Medicine, Department of Infectious Diseases, Division of Internal Medicine, Academic Medical Center, University of Amsterdam, Amsterdam, The Netherlands; Department of Paediatrics and Child Health, Red Cross War Memorial Children’s Hospital (RCWMCH), University of Cape Town, Cape Town, South Africa; Department of Pediatric Pneumology and Immunology, Charité - Universitätsmedizin Berlin, Augustenburger Platz 1, 13353 Berlin, Germany; Institute of Infectious Disease and Molecular Medicine, University of Cape Town, Cape Town, South Africa; Department of Medicine, Klinikum Muenchen-Perlach, Munich, Germany; Division of Infectious and Tropical Diseases, San Matteo Hospital Foundation, University of Pavia, Pavia, Italy; Infectious and Tropical Diseases Unit, San Bortolo Hospital, Vicenza, Italy

**Keywords:** HIV, Pulmonary, Infection, Chest ultrasonography, Diagnostic imaging, Lung disease

## Abstract

**Introduction:**

Pulmonary disease is common in HIV-infected patients. Diagnostic means, however, are often scarce in areas where most HIV patients are living. Chest ultrasonography has recently evolved as a highly sensitive and specific imaging tool for diagnosing chest conditions such as pneumothorax, pneumonia and pulmonary edema in critically ill patients. This article addresses the issue of imaging and differentiating common pulmonary conditions in HIV-infected patients by chest ultrasonography.

**Methods:**

We report chest ultrasound features of five different common pulmonary diseases in HIV-infected patients (bacterial pneumonia, *Pneumocystis jirovecii* pneumonia, tuberculosis, cytomegalovirus pneumonia and non-Hodgkin lymphoma) and review the respective literature.

**Conclusions:**

We observed characteristic ultrasound patterns especially in *Pneumocystis jirovecii* pneumonia and pulmonary lymphoma. Further exploration of chest ultrasonography in HIV-infected patients appears promising and may translate into new diagnostic approaches for pulmonary conditions in patients living with HIV.

**Electronic supplementary material:**

The online version of this article (doi:10.1007/s15010-015-0780-z) contains supplementary material, which is available to authorized users.

## Introduction

Ultrasonography has gained increasing interest during the past two decades. Various medical specialties have adopted focused ultrasound protocols, which are applied by physicians to immediately answer medically important questions, and to guide clinical management [1]. Portable ultrasound machines are available at reasonable cost. Hence, point-of-care ultrasonography is becoming a medical imaging modality suitable for resource-limited settings, where radiological equipment and expertise are scarce.

Chest ultrasonography is among the most recently emerging ultrasonography applications. For years the prevailing opinion was that ultrasound would be unsuitable for diagnosing lung pathologies due to air impeding transmission of sound waves. Today the value of ultrasound in visualizing lung pathologies arising close to the pleura is frequently documented. Assessment with ultrasound has proved sensitive for respiratory conditions such as pneumothorax [2–4], pneumonia [5–11], pleural effusion [12, 13], and pulmonary edema [14, 15], it also decreases time to diagnosis [11], reduces costs [11], and exposure to ionizing radiation [16].

Ultrasound features representing healthy lungs or conversely indicating pathology are well described [17]; assessment of a few sonographic features (A-lines, B-lines, lung sliding, hepatization, anechogenicity) is usually enough to differentiate between healthy lung, consolidation, interstitial disease or pleural disease. In brief, A-lines are horizontal, hyperechoic reverberation artifacts from the pleural line and represent healthy lung parenchyma. B-lines are vertical, hyperechoic reverberation artifacts projecting from the pleural line to the bottom of the screen; occurrence of more than three B-lines in one intercostal space indicates interstitial pathologies of the lung tissue (sensitivity 97 %, specificity 95 %) [18]. Pleural effusions are typically represented by anechoic collections between chest wall and lung. Lung consolidations present as subpleural hypoechoic areas, possibly with hyperechoic air bronchograms. As their echogenicity mimics liver tissue they are described as “hepatization” and can be a feature of infection, malignancy, pulmonary embolism or atelectasis.

Pulmonary diseases are common in HIV-infected patients [19]. Opportunistic infections, neoplastic diseases and other pulmonary pathology occur more frequently than in non-infected individuals [20]. Lung infection due to *Pneumocystis jirovecii*, (*PJP*, previously *P. carinii* pneumonia) was the first opportunistic infection described in HIV-infected patients and the striking increase in incidence furthered the description of the Acquired Immunodeficiency Syndrome (AIDS) [21]. Increasing access to anti-retroviral treatment (ART) has changed the global epidemiology of pulmonary manifestations in HIV, but pulmonary disease still accounts for about one-third of admissions of HIV patients [19]. The burden of pulmonary disease is underlined by the high incidence of pulmonary tuberculosis (PTB) in HIV patients in sub-Saharan Africa [22].

The differential diagnosis of pulmonary disease in HIV-infected patients is broad (see Table 1). History is helpful in the diagnostic process, as is the CD4 count as a marker of immunosuppression. Occurrence of opportunistic infections depends on the degree of immunosuppression; PJP and atypical mycobacterioses mainly occur when CD4 cell counts are <200 cells/ml; CMV and disseminated fungal infections are mainly seen when CD4 counts fall <100 cells/ml [20]. PTB is an infection affecting HIV patients at any stage of the disease with CD4 counts often still being >500/µL.

**Table 1:** Correlation of radiological patterns and ultrasound findings in pulmonary disease in patients with HIV infection (radiological pattern and possible etiology adapted and modified from [58])

Radiological pattern	Expected US pattern	Possible etiology
Without radiological changes	A-lines Pleural line moving normally	PJP Asthma KS of the trachea
Focal infiltrates	Subpleural hypoechoic region ± hyperechoic air bronchograms “Hepatization” of the lung	Bacterial pneumonia Mycobacteriosis Fungi Lymphoma Lung cancer
Interstitial pattern	B-lines Possible small subpleural hypoechoic regions	PJP CMV KS Lymphocytic interstitial pneumonia Interstitial lung diseases Cardiac insufficiency Fungi
Miliary pattern	Not reported	Mycobacteriosis Fungi
Pneumothorax	A-lines Absent lung sliding Identifiable ‘lung point’ In M-mode: “seashore sign”	PJP
Cavernous lesions	Not reported	Mycobacteriosis Bacterial abscess (*Staphylococcus, Pseudomonas*) Lung cancer
Cystic lesions	Multiple small echogenic gas containing lesions surrounded by hypoechoic solid lung	PJP Fungi PTB
Pleural effusion	Anechoic collection between chest wall and the lung Lung tissue may appear echogenic (compression atelectasis) Echogenic fibrin strands and septae possible	Bacterial pneumonia Mycobacteriosis KS Lymphoma Cardiac insufficiency

*US* ultrasound, *PJP* *Pneumocystis jirovecii* pneumonia, *KS* Kaposi’s sarcoma, *CMV* cytomegalovirus, *TB* tuberculosis

For a definitive diagnosis, identification of the pathogen or histo-pathological workup from bronchial secretions or diseased tissue is paramount. The majority of HIV-infected patients live in resource-limited settings, where sputum microscopy for acid-fast bacilli may be the only microbiological test available.

Imaging of the diseased lung can reveal patterns suggesting causative pathogens and processes. Chest radiographs and computed tomography (CT) showing consolidations, ground-glass opacity, cystic lesions or pulmonary nodules permit narrowing of differential diagnosis [23]. Many emergency departments in industrialized countries are equipped with CT, but access to even basic radiological imaging technology is often limited in countries where most HIV-infected patients live [24]. This widespread shortage of imaging services in resource-limited settings significantly reduces health care quality and increases health care disparities [25]. Innovative imaging approaches such as clinician-performed sonography may help to improve the situation [26, 27], provided that availability of ultrasound machines is matched by appropriate and sustained staff training.

Data on chest ultrasonographic findings in HIV-infected patients are very limited. However, as pulmonary diseases do significantly contribute to HIV-related morbidity and mortality, point-of-care chest sonography recommends itself to quickly orient the clinician to differential diagnoses, required investigations and management decisions.

Here we present a case series of HIV-infected patients with typical pulmonary conditions undergoing chest ultrasound in Vicenza, Italy. The patients each had additional standard radiological workup. All ultrasound examinations were performed by MTG, a clinician specialized in infectious diseases and tropical medicine with over 20 years of experience in ultrasound in infectious diseases. Ultrasound examinations were performed using a Aplio XG Model SSA-790A (Toshiba, Tokyo, Japan), with a 3.5-MHz convex probe and an 8-MHz linear probe. The ultrasound studies were performed as point-of-care examination as part of routine clinical care. The convex probe was used first; in the case of superficial or pleural disease, scanning was also performed with the linear probe. Approval for publication was sought by the Ethics Committee for Clinical Research of the Vicenza Province (Comitato Etico per le Sperimentazioni Cliniche della Provincia di Vicenza, approval number 57/2014).

## Case series

### Case 1: Bacterial pneumonia (*Haemophilus influenzae*)

**Fig. 1:**
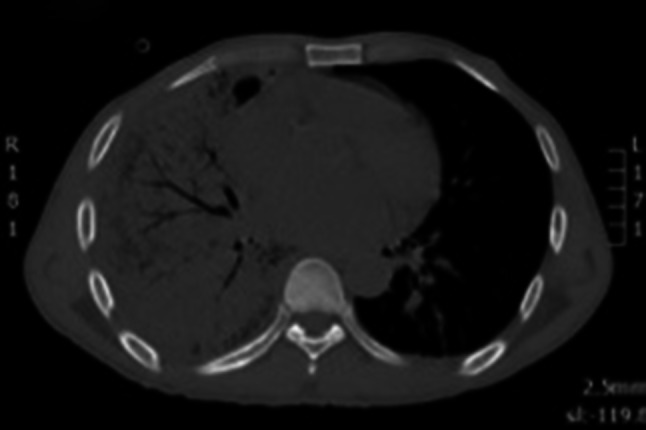
Lung CT scan showing right basal consolidation and air bronchograms

**Fig. 2:**
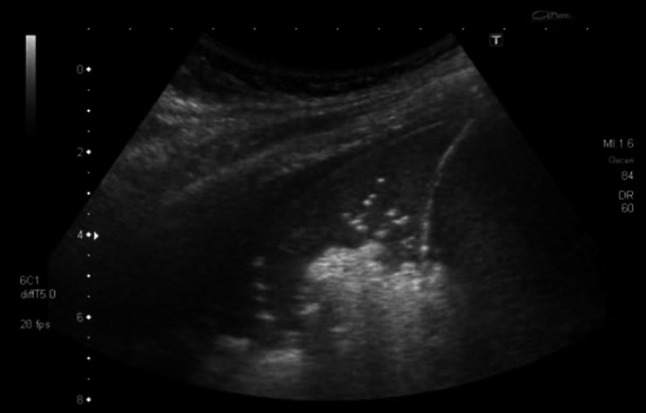
Transthoracic ultrasonography showing a hypoechoic consolidation with string-like echogenic reflexes due to remaining air in the bronchial system (sonographic air bronchogram). Additionally, a small pleural effusion is visible

A 50-year-old HIV-infected man from Burkina Faso, who was non-compliant to ART, presented with fever, chills and chest pain. His CD4 count was 112 cells/μL. On admission, his chest radiograph (Online Resource 8) and CT (Fig. 1) showed pneumonia with pleural effusion. Lung ultrasound performed on the same day revealed a large consolidation with hyperechoic air bronchograms and a small pleural effusion (Fig. 2 and Online Resource 1). While cultures of bronchial secretions remained negative, blood cultures grew *Haemophilus influenzae*. The patient was successfully treated with antibiotics and ART was re-started after recovery.

### Case 2: *Pneumocystis jirovecii* pneumonia

**Fig. 3:**
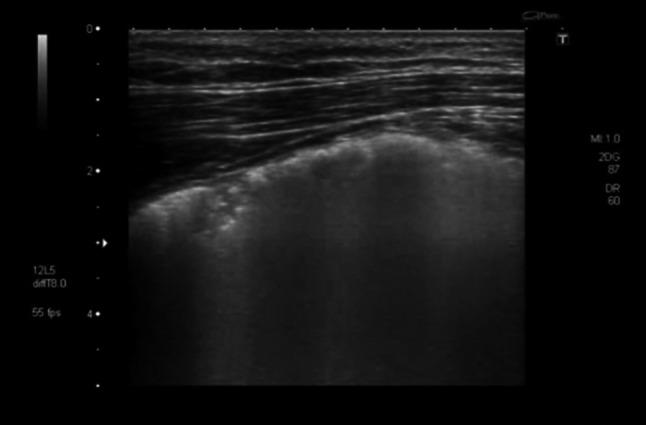
Transthoracic ultrasonography of the pleura shows an interstitial pattern with multiple B-lines. Additionally, small subpleural consolidations are present

**Fig. 4:**
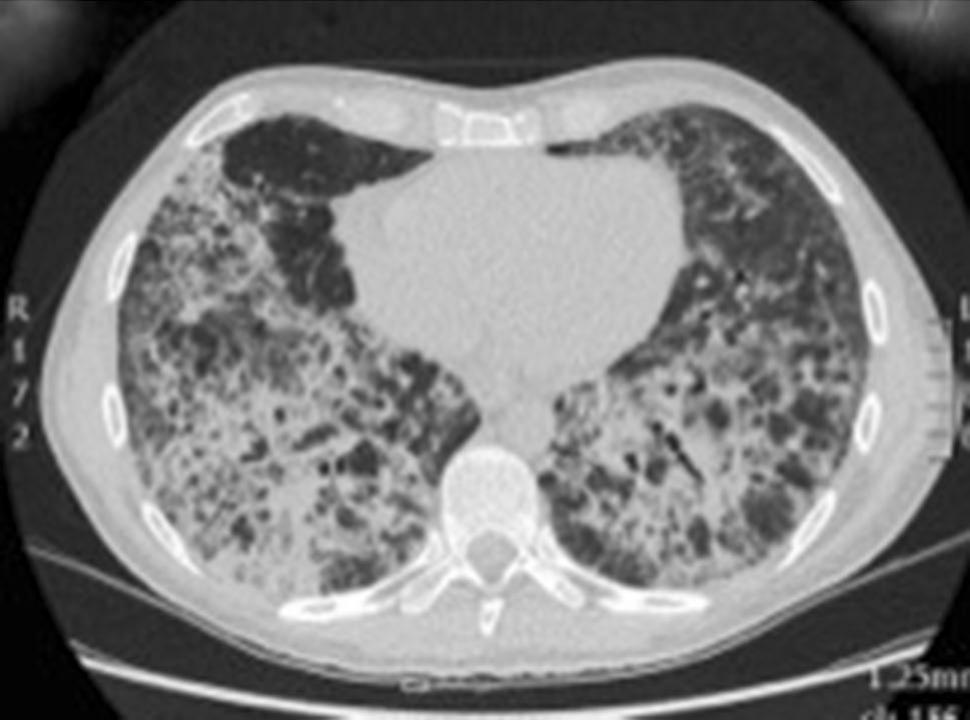
Chest CT after development of respiratory distress showing coarse interstitial thickening with cyst formation and areas of unaffected parenchyma suggestive of pneumocystis pneumonia, which was later microbiologically confirmed in BAL

**Fig. 5:**
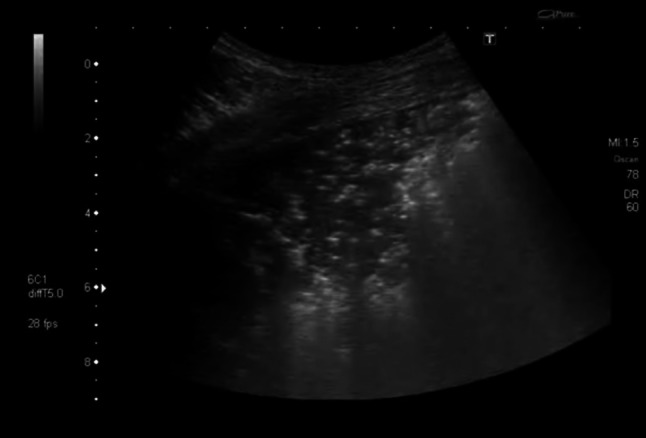
Transthoracic ultrasonography showing large consolidated areas with multiple bright artifacts suggesting gas inclusions. These represent both air bronchograms and cystic areas of the lung

A 35-year-old man from Italy with a history of syphilis presented with fever and weight loss. The patient tested HIV positive; his CD4 count was 34 cell/μL. Initially the patient denied experiencing any respiratory symptoms. His chest radiograph on admission was normal, but lung ultrasound performed on the same day showed multiple B-lines suggesting an “interstitial pattern” of lung injury. Additionally, small peripheral consolidated areas were noted, indicating subclinical lung pathology (Fig. 3 and Online Resource 2). A subsequent chest radiograph (Online Resource 9) and CT scan (Fig. 4) performed 4 days later suggested PJP; BAL microbiologically confirmed this. During the following 10 days he developed respiratory distress. The lung ultrasonography now showed large consolidated areas with bright, hyperechoic reflexes, suggesting gas and fluid trapping (Fig. 5 and Online Resource 3). The patient deteriorated requiring mechanical ventilation, *Pneumocystis jirovecii* pneumonia (PJP) was treated with co-trimoxazole and he slowly recovered.

### Case 3: Tuberculosis

**Fig. 6:**
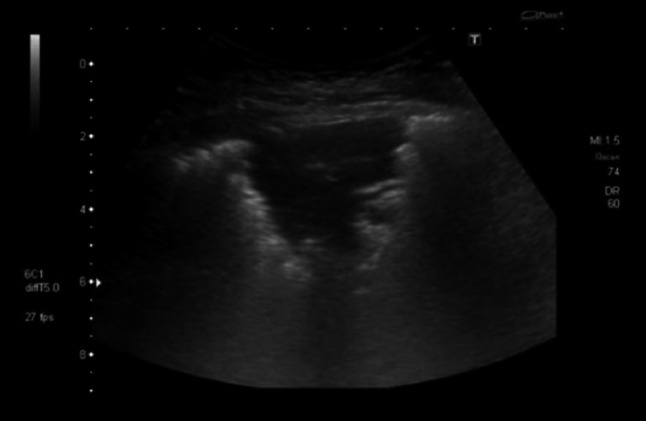
Transthoracic ultrasonography showing a subpleural hypoechoic consolidation. Due to the shredded, fractal boundary between the consolidation and the underlying aerated lung this is called “shred sign”

**Fig. 7:**
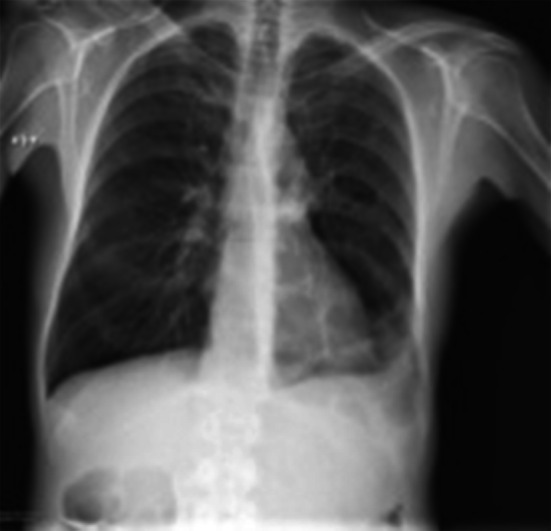
Chest radiograph of a febrile HIV-infected male (CD4 count 173 cells/μl) suggesting a *left* sided basal pneumonia

A 44-year-old Italian drug addict was admitted with fever. Abdominal ultrasound revealed enlarged hypoechoic abdominal lymph nodes and disseminated splenic hypoechoic lesions suggesting micro-abscesses; disseminated TB was suspected. The HIV test was positive and CD4 count was 173 cells/μL. Lung sonography at admission revealed a consolidation in the left lower lobe, resembling a “torn out” area of the lung (“shred sign”) (Fig. 6 and Online Resource 4). This raised suspicion of infiltrative pulmonary involvement by TB disease, which a chest radiograph performed the same day confirmed (Fig. 7); a subsequent BAL showed acid-fast bacilli. The patient received anti-tuberculous treatment and subsequently ART.

### Case 4: Cytomegalovirus pneumonia

A 54-year-old HIV-infected man from Ivory Coast presented with fever, dyspnea, anemia and bloody stools. On admission his CD4 count was 15 cells/μL. On admission a chest radiograph showed a diffuse interstitial pattern (Online Resource 10).

**Fig. 8:**
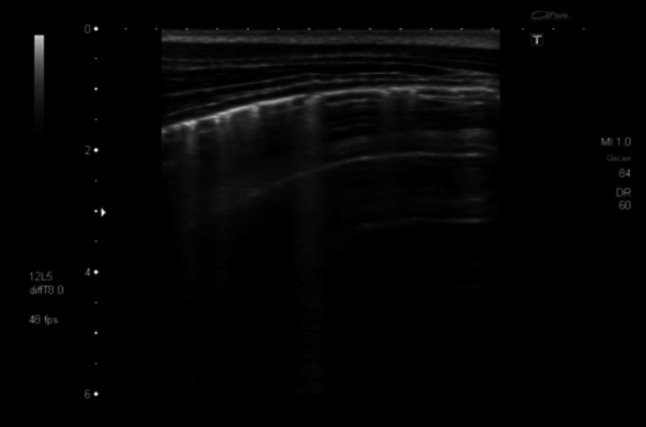
Transthoracic ultrasonography of the pleura. Multiple B-lines are visible suggesting an interstitial pattern of lung injury; as these are not penetrating the complete image, nor do they extinguish the A-lines completely which are visible towards the right side of the screen, these should be referred to as “lung rockets”

**Fig. 9:**
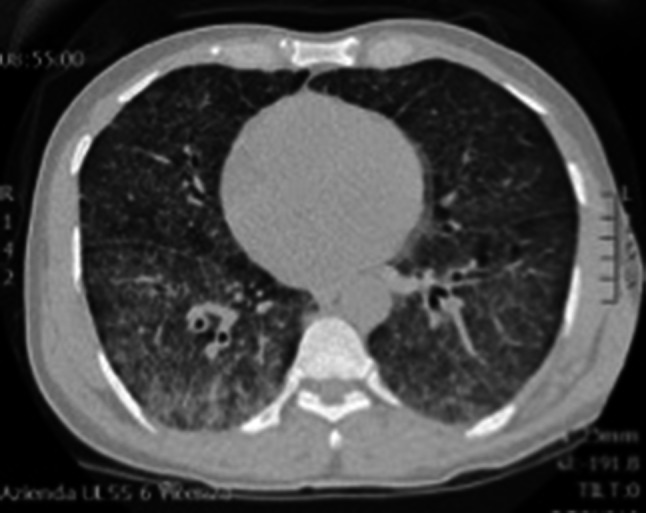
Chest CT scan showing ground-glass appearance and interstitial thickening due to interstitial pneumonia caused by the underlying generalized CMV infection

Lung ultrasonography on the same day showed multiple B-lines originating from the pleura, which did not extend completely to the bottom of the screen (Fig. 8 and Online Resource 5). These sonographic changes prompted the treating clinician to perform a chest CT scan that suggested interstitial pneumonia (Fig. 9). A subsequent colonoscopy showed multiple bleeding ulcers and CMV-DNA was detected in serum. Treatment with gancyclovir was started with a good clinical response.

### Case 5: Non-hodgkin lymphoma

**Fig. 10:**
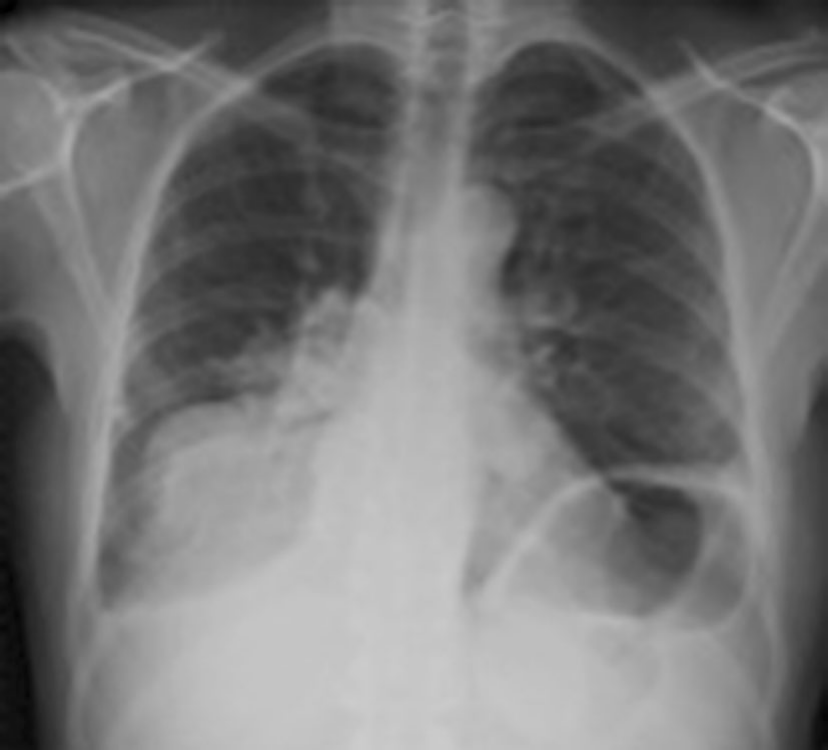
Chest radiograph with consolidation adjacent to the right hemi-diaphragm

A 56-year-old Italian man recently diagnosed with HIV, presented with fever, dyspnea and weight loss. He was non-smoker and worked in the chemical industry. On admission his CD4 count was 53 cells/μL. The chest radiograph performed 8 days after admission showed a dense opacity in the right lower field (Fig. 10); pneumonia and malignancy were suggested as differential diagnoses.

**Fig. 11:**
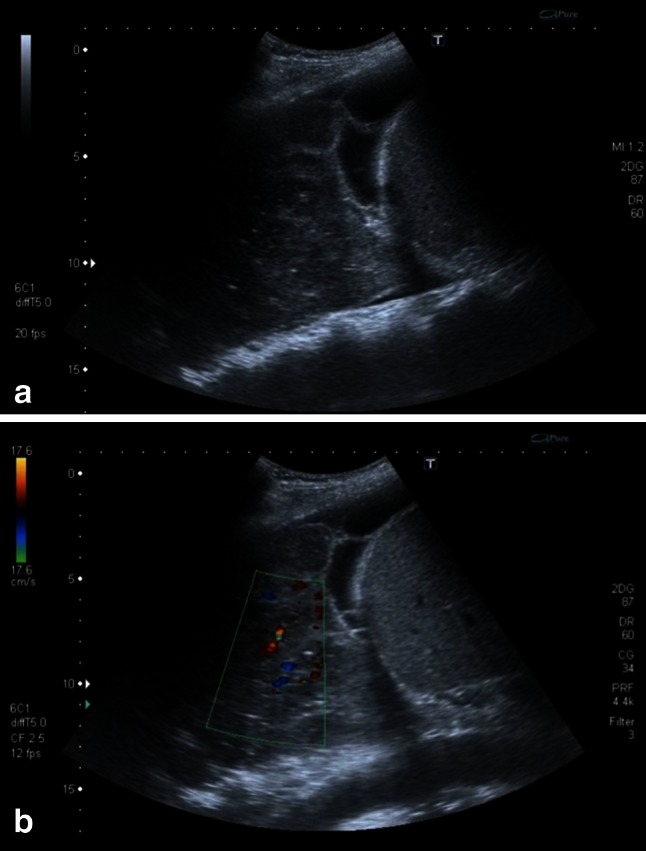
**a** Sonographic image of a right-sided consolidation. The consolidation shows areas of lower echogenicity suggesting lymphoma infiltration. Additionally a pleural effusion with fibrin strands is present. **b** Using color-Doppler the hypoechoic areas surround the vasculature (see Online Resources 6 and 7)

Lung ultrasonography performed on the same day showed a consolidation of the right lower lobe (Fig. 11a and Online Resources 6 and 7). This was not homogenous, suggesting focal parenchymal abnormalities surrounding the vascular structures on color-Doppler (Fig. 11b). Air bronchograms were scarce and only present in the peripheral parts of the consolidation. Retrospectively, the hypoechoic areas were interpreted as lymphoma nodules. A CT-guided lung biopsy (Online Resource 11) revealed an HIV-related diffuse large B cell lymphoma. The patient was treated with poly-chemotherapy (CHOP) and ART and improved.

## Discussion

The cases above describe five HIV-infected patients with different HIV-related pulmonary diseases where clinician-performed chest ultrasound proved useful in the diagnostic workup. The following paragraphs discuss the diseases and the associated radiological and ultrasound patterns (Table 1) and review the literature currently available.

### Bacterial pneumonia

Bacterial pneumonia, predominantly due to *Streptococcus pneumoniae*, *Staphylococcus aureus* and *Haemophilus influenzae* remains a major cause of morbidity and mortality in HIV-infected people [28–30]. Since this condition can present with minimal symptoms in patients with low CD4 cell counts, pneumonia is more likely to be misdiagnosed.

Recently, the use of chest ultrasonography for the diagnosis of pneumonia in adults and children has been studied extensively [5–7, 9–11, 31]. Several studies showed that chest ultrasonography was as sensitive (93–98 %) and specific (95–97 %) as chest radiography in diagnosing pneumonia [5–7, 10, 11, 31]. Hyperechoic air bronchograms in a hypoechoic consolidated area with ill-defined margins are typical sonographic findings in patients with pneumonia (70–97 %) [7–9]. On ultrasonography, affected lung areas appear as a “solid” structure, referred to as hepatization of the lung, which may be accompanied by pleural line attenuation over the affected area. In 34–61 % of the cases these findings are accompanied by pleural effusion, which presents as an anechoic area surrounding the lung tissue [6–9]. To maximize sensitivity with chest ultrasound the whole thorax needs to be scanned by ultrasound circumferentially but still pneumonic lung areas may be missed if ventilated lung parenchyma is interposed; retrocardial or bronchopneumonia thus cannot be ruled out by means of ultrasound.

Our patient’s chest ultrasound findings are similar to those previously described in immune competent patients with a hypoechoic area with air bronchograms and an accompanying pleural effusion.

### *Pneumocystis jirovecii* pneumonia

PJP remains one of the most prevalent opportunistic infection in patients infected with HIV. Patients with CD4 cell counts below 200 cells/µL not receiving prophylaxis are particularly at risk [32]. Common presentation of PJP includes progressive dyspnea, non-productive cough, and fever [33]. Increasing hypoxemia may lead to respiratory insufficiency requiring mechanical ventilation; acute deterioration with chest pain may indicate the development of pneumothorax. PJP may be difficult to diagnose due to non-specific symptoms and signs, which overlap with those of other infections.

PJP causes bilateral interstitial infiltrates. On chest radiography, ground-glass opacity due to fluid, debris and cells in the interstitium and alveoli is present in the majority of patients [34]. Ten to 40 % of PJP patients develop thin-walled cystic lesions within the lung. Little data concerning the use of ultrasound in the diagnosis of PJP exists. In a case description bilateral presence of B-lines is reported, while A-lines remained visible [35]. A small case series of six PJP cases from Brazil found bilateral symmetric B-lines without pleural effusion in all six cases, contrasting with the asymmetric distribution of findings in TB and community acquired pneumonias [36].

We saw B-lines at the margins of the diseased lung parts; additionally, lung ultrasound at a later stage showed a high number of hyperechoic areas within the consolidated lung, which cannot be explained by air bronchograms alone. These echogenic areas did not show shadows. Correlating these focal findings with changes seen in CT scan performed at the same time, we are drawn to conclude that they most likely represent the cystic changes typical for PJP; cystic changes possibly filled with material consisting in gas and fluid. This pattern of consolidation with hyperechoic foci has not been previously described, its frequency and specificity for PJP should be investigated in further prospective studies.

### Tuberculosis

TB remains the most common opportunistic infection in HIV patients worldwide and mortality rates remain high. In 2012 1.1 million HIV-infected patients were newly diagnosed with TB [37]. Patients typically present with cough, weight loss and night sweats/fever, but a significant fraction of patients show mainly constitutional symptoms and less pronounced pulmonary findings, possibly due to accompanying extrapulmonary disease.

Diagnosis of pulmonary tuberculosis by sputum smear microscopy is less sensitive in HIV patients; similarly characteristic cavitary lesions are less commonly seen on chest radiography.

Ultrasonography is an excellent diagnostic tool for extrapulmonary TB in HIV-infected patients, which mostly presents as pleural effusion, pericardial effusion or abdominal TB [38]. Mediastinal ultrasonography for mediastinal tuberculous lymphadenopathy has also been reported [39–41]. Pleural effusions, which appear “complex” on ultrasound, in that they are septated, have fibrin strands or contain echogenic material, are likely to be exudates. TB is the most common cause for exudative effusion in high-prevalence settings [42].

The use of chest ultrasonography to evaluate PTB has not been systematically studied yet; the main radiological features of PTB seen on other imaging modalities such as CT are focal opacities, nodules, cavities and collapsed lung segments or lobes [43], which are potentially visible features on chest ultrasonography as well.

The initial abdominal ultrasound of our patient showed two sonographic features highly suggestive of abdominal TB: abdominal lymphadenopathy and splenic micro-abscesses. In this context our patient’s pulmonary changes on chest ultrasonography pointed towards pulmonary involvement of TB, which was confirmed by BAL. Consolidations and pleural effusions seen on chest ultrasonography should always include TB in the differential diagnosis.

### Cytomegalovirus pneumonia

Global seroprevalence of CMV is high and reactivation of CMV leading to CMV viremia and disseminated CMV disease is frequent in patients with advanced HIV [44]. CMV retinitis is the most commonly reported CMV localization in HIV-infected patients, but other end-organ diseases such as CMV pneumonitis or CMV colitis are also common and are probably underreported due to difficulties in confirming the diagnosis [45].

Diagnosis of CMV pneumonitis is challenging, but a timely diagnosis and effective treatment are crucial to yield favorable outcomes, especially in severely immunosuppressed HIV patients.

While imaging features of CMV pneumonitis have been described for chest radiography studies and CT scanning, we are not aware of any report on lung ultrasound features of CMV lung disease having been published to date. Findings on chest radiographs and CT are usually non-specific and diverse and include ground-glass opacities, small pulmonary nodules with bilateral distribution involving all zones; confluent consolidation may be more marked towards the lower lobes [46–50]; the differential diagnosis includes other viral pneumonias and PJP [51].

Chest ultrasound features seen in our patient with CMV pneumonitis are in line with the expected patterns of interstitial alteration represented by B-lines. In our patient with CMV pneumonitis the B-lines were narrower and did fade in the deeper regions on the screen, making them less pronounced than the B-lines seen in the patient with PJP; furthermore, the interstitial pattern seen in CMV pneumonitis did not show any air inclusions or consolidations observed in the PJP pattern. Whether these differences in interstitial lung ultrasound pattern represent sufficiently typical characteristics of PJP and CMV lung disease, respectively, and may therefore serve as diagnostic differentiators, needs to be addressed in further studies.

### AIDS-related lymphoma (ARL)

ARL is the second most common neoplastic disease associated with HIV infection; the risk of developing non-Hodgkin lymphoma within 3 years of AIDS diagnosis is 165-fold higher than in people without AIDS [52]. Most ARL are high-grade B cell lymphomas and a role of Epstein-Barr-Virus infection in the pathogenesis is hypothesized [53]. ARL tends to be highly aggressive, presents at a late stage and extra-nodal involvement is seen in up to 90 % [54]. The central nervous system, gastrointestinal tract and liver are commonly affected sites. Lung involvement is not uncommon, in autopsy series the lung was the most frequent extra-nodal site of ARL disease [55].

Patients with pulmonary ARL present most commonly with cough and dyspnea [55]; additionally, constitutional B-symptoms are prevalent. Most patients present with hilar lymphadenopathy or enlarged lymph nodes elsewhere; primary pulmonary ARL exists but is rare [23].

Pulmonary nodules, lobar infiltrates and lung masses are the most common parenchymal abnormalities [55]. Unilateral or bilateral effusions are found in around 50 % of cases and may be the only manifestation of pulmonary ARL. To our knowledge, data on the sonographic appearance of pulmonary ARL has not been reported so far, while ultrasound findings of abdominal ARL have been well described. On ultrasonography most ARL cases affecting solid organs like liver or spleen, present as hypoechoic nodules, which are normally multiple and range in size from 0.5 to 10 cm [56]. A special pattern of lymphoma involvement in the liver is periportal infiltration; in these cases, ARL appears as hypoechoic confluent masses, which are localized around intrahepatic vessels [57].

In our patient sonographic hypoechoic nodules can be distinguished within echogenic, consolidated lung. Similar to hepatic cases the masses appear to encase or ‘follow’ the vasculature of the lung. This nodularity observed in our case suggests a sonographic pattern slightly different from more homogenous pneumonic infiltrates; the specificity of this pattern cannot be commented on from this single case and further cases need to be studied.

## Conclusion

Chest ultrasonography is a valuable diagnostic tool for a variety of respiratory infections and diseases as demonstrated here in HIV-infected patients. The five cases described represent common respiratory conditions in HIV-infected patients and sonographic patterns suggestive of the conditions were identified. However, at this point the specificity of the ultrasound findings cannot be generalized and further systematic studies in patients with these pulmonary conditions are needed. Among the many benefits related to ultrasound are its low costs, radiation-free imaging, real-time imaging, its mobility, repeatability and suitability for remote settings, where many patients with severe conditions are seen but imaging modalities are lacking. The minimal technical requirements of the equipment needed for lung assessment should be further studied, although in our opinion and experience basic portable ultrasound machines with both, convex and linear probes seem adequate. Albeit ultrasonography can only identify pathological tissue patterns and not the underlying etiology, differential diagnosis can be narrowed, allowing more focused action, reduced time to diagnosis, reduced costs and ultimately better outcomes.

## Electronic supplementary material


**Online resource 1** Transthoracic ultrasonography of the lung showing a large consolidation containing string-like echogenic reflexes representing pneumonia and air bronchogram (M4 V 6858 kb)

**Online resource 2** Transthoracic ultrasonography shows multiple echogenic B-lines moving with respiration. Additionally, small (less than 1 cm) consolidations right below the pleura are visible intermittently (M4 V 4122 kb)

**Online resource 3** Transthoracic ultrasonography after clinical deterioration due to pneumocystis pneumonia. A large hypoechoic, consolidated area of lung is visible. Within this area multiple bright, echogenic artifacts are seen, representing both intrabronchial air and cystic changes of the lung (M4 V 8740 kb)

**Online resource 4** Transthoracic ultrasonography of the lung showing a sub-pleuralhypoechoic region with a tissue like texture suggesting an infiltrate. Due to the shredded, fractalboundary between the consolidation and the underlying aerated lung this is called “shred sign” (M4 V 6306 kb)

**Online resource 5** Transthoracic ultrasonography of the pleura. Multiple vertical lines are visible suggesting an interstitial pattern of lung injury. Towards the right side, normal horizontal A-lines are visible. As the vertical lines are not penetrating the complete image and do not extinguish the A-lines completely they are called “lung rockets” suggesting a less severe lung injury compared to B-lines (M4 V 5242 kb)

**Online resource 6** Transthoracic ultrasonography of a right lung consolidation. On the right side of the screen, liver, kidney and diaphragm are visible. Above the diaphragm (to the left) a black effusion with echogenic fibrin strands surrounding consolidated lung is seen. Using the color-doppler mode the spacial relation of the hypoechoic areas and the vasculature is visible (toward the end of the clip) (M4 V 10721 kb)

**Online resource 7** In sub-costal ultrasonography the inhomogeneous lung consolidation is seen behind the liver and diaphragm. Again the “nodularity” is well visible (M4 V 4867 kb)

**Online resource 8** Chest radiograph suggesting pneumonia and effusion on the right side (JPEG 83 kb)

**Online resource 9** Chest radiograph after development of respiratory distress. Ground-glass opacity suggested pneumocystis pneumonia, which was later microbiologically confirmed in BAL (JPEG 67 kb)

**Online resource 10** Chest radiograph on admission upon fever and dyspnea. Mild diffuse interstitial changes were reported (JPEG 73 kb)

**Online resource 11** CT guided biopsy of a dorsal right lung consolidation; histology revealed a HIV related B-cell lymphoma (JPEG 55 kb)

## Abbreviations

AIDS: Acquired immunodeficiency syndrome
ART: Anti-retroviral therapy
BAL: Bronchoscopic alveolar lavage
CHOP: Cyclophosphamide, hydroxydaunorubicin, oncovin and prednisolone
CMV: Cytomegalovirus
CT: Computed tomography
DNA: Deoxyribonucleic acid
HIV: Human immunodeficiency virus
MRI: Magnetic resonance imaging
PJP: *Pneumocystis jirovecii* pneumonia
PTB: Pulmonary tuberculosis
TB: Tuberculosis
