# Supplementary figures and images for: Chest ultrasonography in patients with HIV: a case series and review of the literature
Source: Infection. 2015 May 14;44:1–10. doi: 10.1007/s15010-015-0780-z (PMC4735240; doi:10.1007/s15010-015-0780-z)

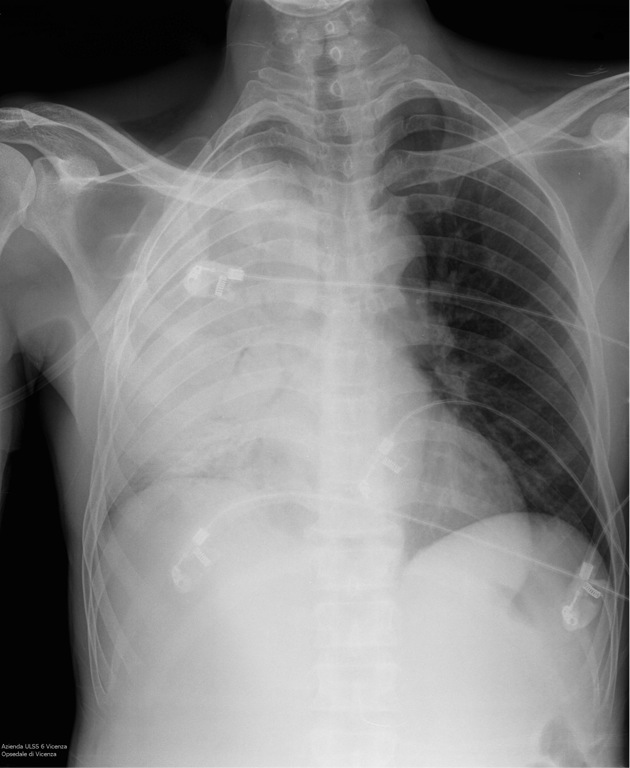

Supplement: Supplementary file 8 — Online resource 8 Chest radiograph suggesting pneumonia and effusion on the right side (JPEG 83 kb) [file 15010_2015_780_MOESM8_ESM.jpg]

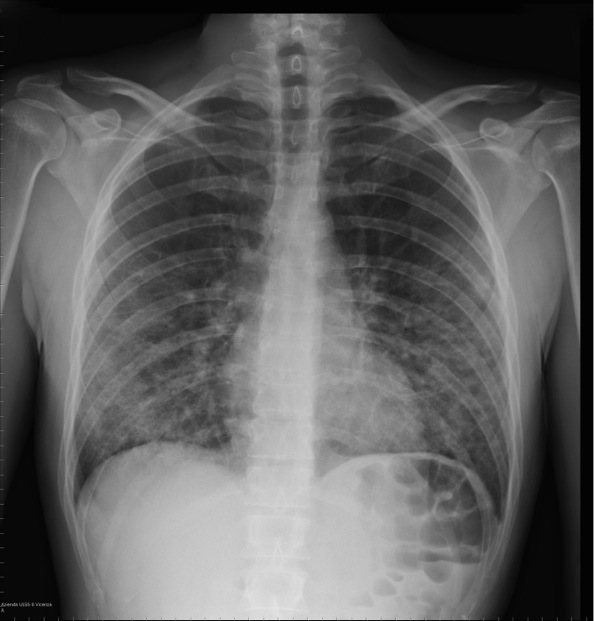

Supplement: Supplementary file 9 — Online resource 9 Chest radiograph after development of respiratory distress. Ground-glass opacity suggested pneumocystis pneumonia, which was later microbiologically confirmed in BAL (JPEG 67 kb) [file 15010_2015_780_MOESM9_ESM.jpg]

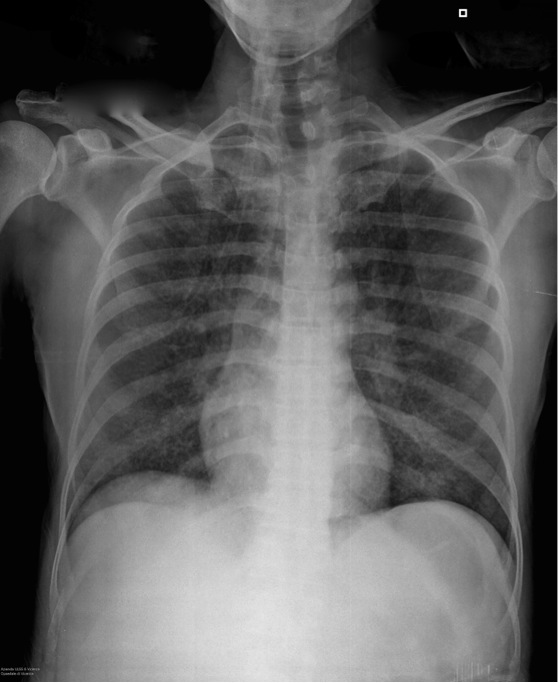

Supplement: Supplementary file 10 — Online resource 10 Chest radiograph on admission upon fever and dyspnea. Mild diffuse interstitial changes were reported (JPEG 73 kb) [file 15010_2015_780_MOESM10_ESM.jpg]

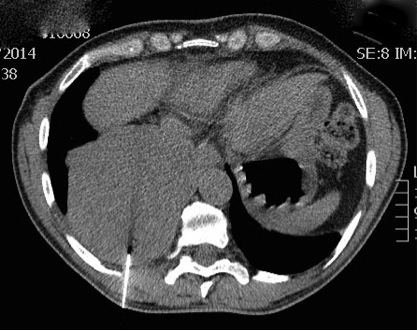

Supplement: Supplementary file 11 — Online resource 11 CT guided biopsy of a dorsal right lung consolidation; histology revealed a HIV related B-cell lymphoma (JPEG 55 kb) [file 15010_2015_780_MOESM11_ESM.jpg]
